# Reinfection-Driven Accumulation of SARS-CoV-2 Antibodies: A 36-Month Longitudinal Study in Austrian Blood Donors

**DOI:** 10.3390/diagnostics16020195

**Published:** 2026-01-08

**Authors:** Orkan Kartal, Alexandra Domnica Hoeggerl, Wanda Lauth, Lisa Weidner, Natalie Badstuber, Christoph Grabmer, Christof Jungbauer, Verena Nunhofer, Heidrun Neureiter, Nina Held, Tuulia Ortner, Eva Rohde, Sandra Laner-Plamberger

**Affiliations:** 1 Department of Transfusion Medicine, University Hospital of Salzburg (SALK), Paracelsus Medical University (PMU) Salzburg, Müllner-Hauptstraße 48, 5020 Salzburg, Austria; o.kartal@salk.at (O.K.); a.hoeggerl@salk.at (A.D.H.); lisa.weidner@roteskreuz.at (L.W.); c.grabmer@salk.at (C.G.); christof.jungbauer@roteskreuz.at (C.J.); h.neureiter@salk.at (H.N.); n.held@salk.at (N.H.); e.rohde@salk.at (E.R.); 2 Team Biostatistics and Big Medical Data, IDA Lab Salzburg, Paracelsus Medical University (PMU) Salzburg, Strubergasse 21, 5020 Salzburg, Austria; wanda.lauth@pmu.ac.at; 3 Research Programme Biomedical Data Science, Paracelsus Medical University (PMU) Salzburg, Strubergasse 21, 5020 Salzburg, Austria; 4 Austrian Red Cross, Blood Service for Vienna, Lower Austria and Burgenland, Wiedner Hauptstraße 32, 1040 Vienna, Austria; verena.nunhofer@roteskreuz.at; 5 Department of Psychological Assessment, Institute of Psychology, Paris-Lodron-University of Salzburg, 5020 Salzburg, Austria; natalie.badstuber@plus.ac.at (N.B.); tuulia.ortner@plus.ac.at (T.O.); 6 GMP Laboratory, PMU Salzburg, Strubergasse 21, 5020 Salzburg, Austria

**Keywords:** SARS-CoV-2, seroprevalence, anti-N antibodies, blood donation, reinfection

## Abstract

**Background/Objectives**: Long-term serological studies are essential to understand how repeated antigenic exposure affects the specific humoral immune response. The aim of this study was to investigate the long-term SARS-CoV-2 antibody dynamics in Austrian blood donors, as representatives of healthy adults, over a period of 36 months after the first SARS-CoV-2 infection. **Methods**: SARS-CoV-2 anti-N antibody levels were determined in more than 146,000 blood donations collected between 2020 and 2025. In addition, SARS-CoV-2 anti-N and anti-S antibody dynamics were examined in 204 individual blood donors at predefined points in time over a period of 36 months. Reinfections were inferred from increases in anti-N levels within an individual. Vaccination history and self-reported infection data were documented. **Results**: Anti-N seroprevalence was over 90% from the beginning of 2023 and remained at this level until 2025. Among the longitudinally observed participants, 97% had at least one serologically detected reinfection and 50% had two or more. While anti-N levels continued to increase over time, suggesting cumulative antigenic stimulation, anti-S concentrations and in vitro antibody functionality remained consistently high. Self-reported reinfections underestimated the actual incidence by a factor of six. Symptom profiles shifted toward mild respiratory manifestations, with significantly fewer cases of hyposmia or dysgeusia reported compared to the initial infection. **Conclusions**: After three years of observation, SARS-CoV-2 immunity is characterized by sustained antibody activity. The results show a transition from persistent, but inherently declining, to a repeatedly rebuilding, enhanced humoral immunity, indicating that SARS-CoV-2 has become endemic in Austria.

## 1. Introduction

Despite intensive efforts worldwide, through a range of measures such as lockdowns, social distancing and vaccinations, the global spread of the severe acute respiratory syndrome coronavirus-2 (SARS-CoV-2), the causing agent of Coronavirus Disease 2019 (COVID-19), could not be prevented. Although the WHO declared the end of the pandemic in May 2023 (https://www.who.int/news/item/05-05-2023-statement-on-the-fifteenth-meeting-of-the-international-health-regulations-(2005)-emergency-committee-regarding-the-coronavirus-disease-(COVID-19)-pandemic, accessed 7 November 2025), there are still many reinfections, especially with new subvariants of SARS-CoV-2, including the highly infectious omicron variant [1]. To date, there have been 778,856,342 reported cases of COVID-19 worldwide since recording began, with 147,208 new cases being reported to the World Health Organization (WHO) in October 2025 (https://data.who.int/dashboards/covid19/cases, last accessed 7 November 2025). Furthermore, the European Respiratory Virus Surveillance Summary provided by the European Centre for Disease Prevention and Control (ECDC) states that SARS-CoV-2 is currently widespread and elevated among all age groups (https://erviss.org/, last accessed 7 November 2025).

Since the emergence of SARS-CoV-2, the characterization of the specific humoral immunity response following infection and also vaccination has been a major research target. The duration, magnitude and functionality of SARS-CoV-2 antibodies determine not only individual protection against reinfection but also the long-term dynamics of viral circulation and the emergence of new virus variants [2,3,4]. Several studies have shown that infection with SARS-CoV-2 induces an antibody response targeting both the viral spike (S) and nucleocapsid (N) protein, which can be detected for up to one year after infection [5,6,7,8,9]. However, the longevity and dynamics of SARS-CoV-2-specific antibodies in the post-pandemic phase, in which there are still a large number of infections, are not yet fully understood.

In a previous study, we showed with a cohort of Austrian blood donors that within the first year after a SARS-CoV-2 infection, anti-N and anti-S antibodies remained detectable and functional, but levels were significantly declining [6]. In our two-year follow-up, we detected substantially increasing anti-N antibody levels, which are mainly produced after an infection, but not after vaccination, thus suggesting a high reinfection frequency [10]. So far, our data point toward a shift in SARS-CoV-2 antibody kinetics: from primary persistence with detectable, but significantly declining, levels within one year after the initial infection to more complex dynamics, due to repeated antigenic stimulation. A similar finding was reported for US blood donors, where serial samples exhibited boosting of both anti-N and anti-S antibodies [11]. A solid understanding of the SARS-CoV-2 reinfection dynamics in the post-pandemic era is crucial for several reasons. It determines the duration of population immunity; helps us to assess immune escape by emerging variants; provides insights into clinical severity; supports more accurate forecasting of future waves and long-term transmission dynamics, thus increasing the preparedness for future viral outbreaks; and finally, it improves the interpretation of serological data and antibody kinetics. The present study aimed to examine the long-term SARS-CoV-2 humoral immune response, also including a period during the post-pandemic phase from May 2023 until March 2025 in Austrian blood donors, who can be considered representatives of the healthy adult population. In order to do so, we used two datasets: (1) a large cross-sectional dataset consisting of 146,148 blood donor samples collected between June 2020 and March 2025 and (2) a longitudinal panel of 204 participants who donated blood samples after a first SARS-CoV-2 infection at predefined points in time, covering 36 months post-initial seropositive SARS-CoV-2 blood donation, to characterize long-term serological kinetics in more detail.

## 2. Materials and Methods

### 2.1. Ethical Statement

Human residual serum of standard blood donations, which is used for routine laboratory diagnostics of blood product processing according to European and local regulations, was used for the present study. All donors signed informed consent on the use of leftover material for research purposes and all samples were processed anonymously to protect donor privacy. This study was approved by the ethical committee of the Federal State of Salzburg, Austria (reference number 1004/2021, date of approval: 18 February 2021). All work described has been carried out in accordance with the 1964 Helsinki Declaration and its later amendments.

### 2.2. Sample Collection, Study Design and Cohort Characteristics

**Figure 1:**
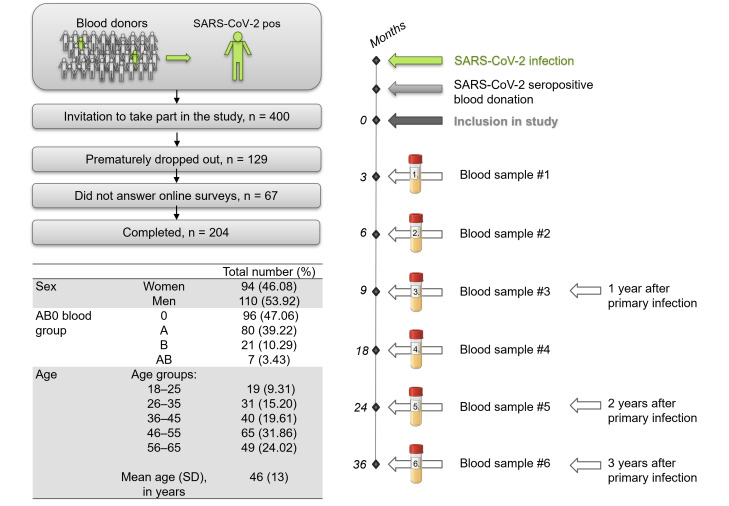
Workflow and cohort characteristics. More than 40,000 blood donors were screened between March 2021 and June 2022. Individuals who screened positive for SARS-CoV-2 anti-N antibodies were invited to take part in the prospective long-term study: 400 individuals were included. Blood samples were collected at 3, 6, 9, 18, 24 and 36 months after the initial SARS-CoV-2 anti-N seropositive blood donation (0 months), while online surveys were to be filled in at 3, 9, 18, 24 and 36 months. A total of 204 individuals completed the study after 3 years and the corresponding data sets were used for further analysis.

To determine the SARS-CoV-2 anti-N seroprevalence rate between June 2020 and March 2025, a total of 146,148 blood donations from over 40,000 individual blood donors were screened. As previously described [6,10], all blood donors underwent a brief health screening prior to admission for blood donation. After donors’ written informed consent for pathogen screening and the use of leftover material for research purposes, blood donations were screened for SARS-CoV-2 anti-N. Due to legal requirements, individuals younger than 18 or older than 70 were not admitted for regular blood donation and were therefore not included. Between March 2021 and June 2022, individuals whose blood donations had previously tested positive for SARS-CoV-2 anti-N were invited to participate in the prospective long-term part of this study. A total of 400 individuals were included, with all data being processed with compliance with the General Data Protection Regulation (GDPR). As described previously, the distribution regarding the sex, ABO blood group and age of this cohort is typical for Austrian blood donors [6]. Known pre-existing conditions were limited to those that do not lead to exclusion from blood donation under applicable national and international regulations, such as non-acute orthopedic conditions or allergies. Individuals who have been deemed ineligible to donate blood for specific health reasons, including, but not limited to, a body weight below 50 kg, cancer, infectious diseases (e.g., HIV, HCV, HBV, Lyme disease, toxoplasmosis and tuberculosis), autoimmune diseases, diseases that require the intake of certain medication (e.g., antibiotics, isotretinoin), drug and alcohol abuse and staying in malaria areas, were not included in the study. It can therefore be assumed that the study cohort consisted of healthy adults. Over the course of the study, participants were asked to provide 6 blood samples (3, 6, 9, 18, 24 and 36 months after the initial anti-N seropositive blood donation) and to fill in 5 online surveys (at 3, 9, 18, 24 and 36 months), which were administered using Lime Survey (LimeSurvey GmbH, Hamburg, Germany, https://www.limesurvey.org, last accessed 2 November 2025), as described [6,10]. Online surveys covered questions regarding the infection, symptoms, vaccinations and reinfections. A total of 129 individuals dropped out prematurely, 67 did not answer the online surveys and 204 participants completed the study (all blood samples requested were provided and online surveys were filled in). These complete data sets were used for further analysis. Figure 1 depicts a graphical summary of the study workflow and the cohort characteristics. It is important to note that the study cohort consists of vaccinated and unvaccinated individuals. Participants were regarded as vaccinated if at least one vaccination was administered (= basic vaccination). Three different vaccines were used: Spikevax^®^ (Moderna, Cambridge, MA, USA) Vaxzevria^®^ (AstraZeneca, Cambridge, UK) and Comirnaty^®^ (BioNTech, Mainz, Germany) as provided by national health services. Some individuals also showed mixed administration of different vaccines. At the end of this study, after 36 months, 158 participants were vaccinated, while 46 participants were never vaccinated (Table A1).

### 2.3. Serological Screening Assays

Three different serological screening methods were employed: (1) the semi-quantitative Elecsys Anti-SARS-CoV-2 (ACOV2) total antibody electrochemiluminescence immunoassay (ECLIA, Roche Diagnostics, Basel, Switzerland), (2) the quantitative ECLIA Elecsys Anti-SARS-CoV-2 S (Roche Diagnostics, Basel, Switzerland) and (3) the SARS-CoV-2 surrogate Virus Neutralization Test (sVNT) (GenScript, Piscataway Township, NJ, USA). As previously described [6,10,12], the ACOV2 ECLIA was used to screen for SARS-CoV-2 anti-N total antibody (IgM, IgG and IgA) using a cobas8000-e801 device (Roche Diagnostics, Basel, Switzerland), according to the manufacturer’s instructions. SARS-CoV-2 anti-N antibodies are produced after a SARS-CoV-2 infection and the levels are expected to be largely unaffected by vaccinations. According to the manufacturer, this screening assay does not discriminate but detects antibodies against all SARS-CoV-2 variants known so far. The quantitative ECLIA Elecsys Anti-SARS-CoV-2 S, which was also conducted on a cobas8000-e801 device, was applied to determine SARS-CoV-2 anti-S antibody (IgM, IgA and IgG) concentrations in IU/mL. Samples that could not be accurately determined due to the detection limits of the assay were diluted 1:100 and measured again. Following the manufacturer’s instructions, the sVNT was applied to investigate the in vitro neutralizing potential of the SARS-CoV-2 antibodies present in the participants’ serum. This was accomplished by quantifying the extent to which the antibodies prevented the human angiotensin-converting enzyme 2 (ACE2) receptor from interacting with a recombinant viral receptor-binding domain. Results are expressed as signal inhibition, given in percent. According to the manufacturer, inhibition rates ≥ 30% should be considered to indicate the presence of functional antibodies.

We calculated the proportion of study participants who had an increase in anti-N antibody levels of at least 30% compared to their previously decreasing values, to determine the reinfection rate based on the serological profile of anti-N antibody levels. Two factors led to the selection of the >30% cut-off: (1) It was shown that six months following antigen contact, SARS-CoV-2 antibody maturation is finished [13]. Consequently, it is unlikely that antibody maturation is the cause of subsequent increases in anti-N levels after this time, which corresponds to 3–6 months in our present study. (2) In another study, by applying ten distinct serological assays, including the Elecsys ACOV2 assay, it was demonstrated that SARS-CoV-2 antibody levels dropped during the first few months after infection but stabilized at about 30% of the peak level observed [8].

### 2.4. Statistical Analysis

Continuous variables are reported as the mean (standard deviation), whereas categorical variables are presented as absolute counts (percentages). Changes between two times (paired) were assessed using the non-parametric Wilcoxon signed-rank test, while differences between groups at a given point in time were evaluated with the Wilcoxon rank-sum test. A two-sided *p*-value < 0.05 was considered to be statistically significant. Line charts with local regression smoothing were used to illustrate the temporal development of the measured variables and trends between groups. Bar and pie charts were employed to depict the relative frequencies of categorical variables. A linear mixed model was calculated to assess the longitudinal development of SARS-CoV-2 anti-N and anti-S antibodies, as well as their in vitro functionality, for 36 months after the initial seropositive blood donation, according to their vaccination status [14]. The model included time and vaccination status as fixed effects to estimate the overall temporal trends and group differences. The participant’s unique identifier number was included as a random intercept to account for repeated measurements within individuals. Interaction terms between time and vaccination status were included to evaluate whether the temporal trajectory of antibodies differed between vaccinated and unvaccinated participants. Model estimates were reported with corresponding standard errors, t-values and *p*-values, which were calculated using Satterthwaite’s degrees of freedom method. To visualize the results, predicted marginal means of antibody levels and in vitro functionality over time for each group were plotted, using line charts with asymptotic 95% confidence intervals, illustrating both the overall trends and group-specific developmental course. All statistical analyses were performed using R (version 4.3.2, R-Core Team, Vienna, Austria) [15].

## 3. Results

### 3.1. SARS-CoV-2 Has Become Endemic in Austria with a Seroprevalence Rate Exceeding 90% in Blood Donors from 2023 Onward

**Figure 2:**
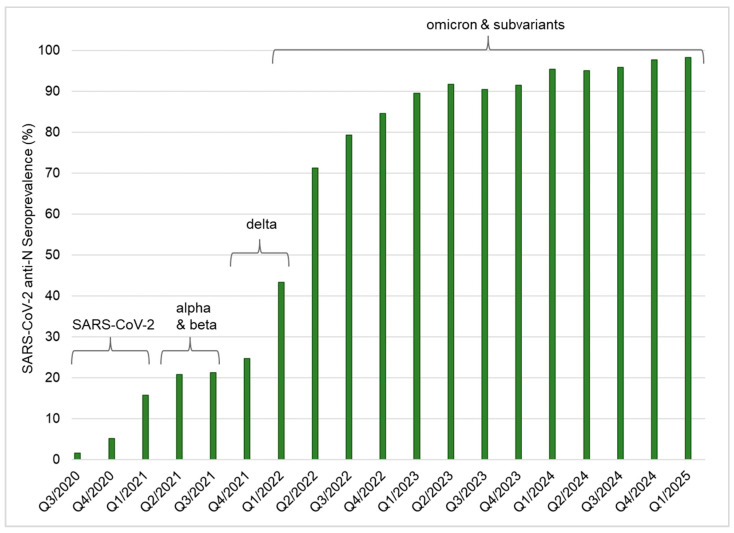
SARS-CoV-2 anti-N seroprevalence in 42,459 blood donors in Salzburg, Austria. A total of 146,148 blood donations were screened for SARS-CoV-2 anti-N antibodies between June 2020 and March 2025: the anti-N seroprevalence is shown in percent. Time is indicated as 1st to 4th annual quarter (Q1-Q4) for each year. SARS-CoV-2 and the most prominent virus variants (alpha, beta, delta and omicron, including its subvariants) circulating in Europe at specific times are indicated, based on the World Health Organization’s (WHO) Coronavirus disease (COVID-19) Epidemiological Updates https://www.who.int/emergencies/diseases/novel-coronavirus-2019/situation-reports (last accessed 3 October 2025).

As already described in our previous studies [6,10,12], SARS-CoV-2 anti-N seroprevalence has been constantly rising since June 2020 in the Federal State of Salzburg, Austria. Figure 2 shows the SARS-CoV-2 anti-N seroprevalence rates of all blood donations in Salzburg between June 2020 and March 2025 (*n* = 146,148). The seroprevalence rate among blood donors increased sharply, especially since the emergence of the SARS-CoV-2 omicron strain and its subvariants, reaching the 90% mark at the beginning of 2023. Until the end of this general blood donor screening in March 2025, the seroprevalence rate had not fallen below 90% (Figure 2).

### 3.2. A Total of 97% Experienced at Least One SARS-CoV-2 Reinfection, 50% Had Two or More Reinfections

**Figure 3:**
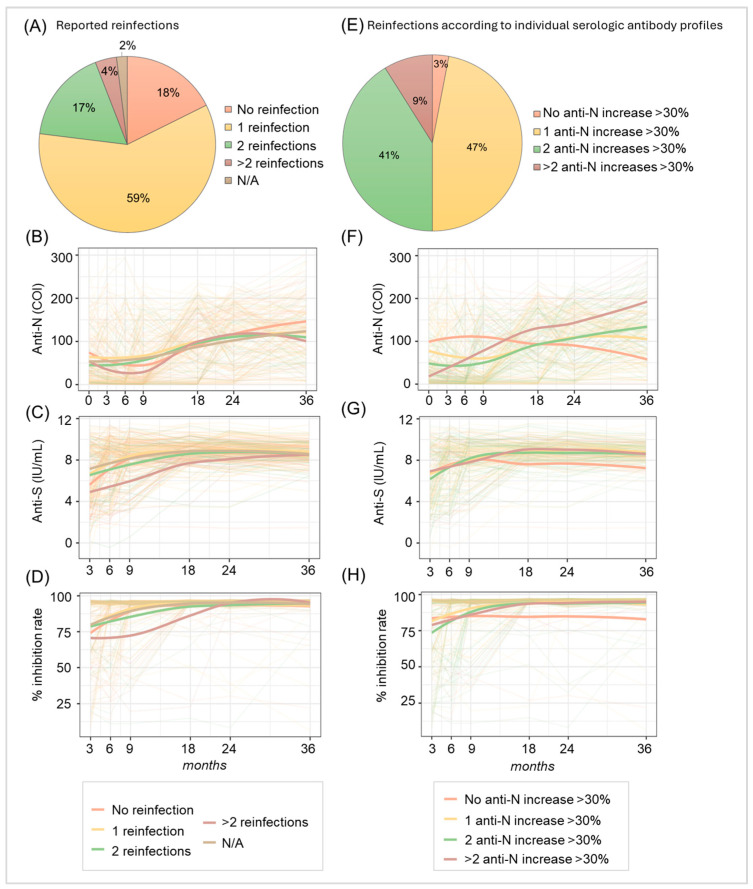
Reported and perceived SARS-CoV-2 reinfection rates 36 months after the initial SARS-CoV-2 seropositive blood donation (*n* = 204). (**A**) Number of reported reinfections by participants. N/A no answer. (**B**) Individual dynamics and local regression of SARS-CoV-2 anti-N antibodies (cut off-indices, COI), (**C**) anti-S antibodies (IU/mL, logarithmic scale) and (**D**) in vitro antibody functionality (in % inhibition rate), according to participants’ reports concerning SARS-CoV-2 reinfections. (**E**) Participants grouped according to their individual serological SARS-CoV-2 anti-N antibody profile (with respect to levels increasing >30% compared to previous points in time). (**F**) Individual antibody dynamics and local regression of anti-N total antibodies (COI), (**G**) anti-S antibody levels (IU/mL, logarithmic scale) and (**H**) in vitro antibody functionality (in % inhibition rate) according to anti-N antibody dynamics, as indicated. The time course shown covers the seropositive SARS-CoV-2 blood donation (0 months) to 36 months thereafter for anti-N antibodies (**B**,**F**) and 3–36 months after the seropositive SARS-CoV-2 blood donation for anti-S antibodies (**C**,**G**) and in vitro antibody functionality (**D**,**H**).

For a more detailed examination, the SARS-CoV-2 antibody dynamics of 204 voluntary blood donors, who were screened as positive for SARS-CoV-2 anti-N antibodies between March 2021 and June 2022, were determined at specific times over a period of 36 months. As described previously [10], we observed high reinfection rates, particularly for the omicron variant, with 59% of the participants reporting a reinfection, while 88% actually experienced at least one. After 36 months, we compared antibody dynamics according to participants’ reports and according to the actual serological profile of anti-N levels again. As before, an increase in anti-N antibody levels of >30% compared to previous values was assumed as an indicator for a reinfection [8,10]. We identified an even higher discrepancy between reported and experienced reinfections compared to our previous results: while 18% of participants reported no reinfections, 59% reported one, 17% stated two reinfections, 4% reported more than two reinfections and 2% did or could not answer this question (Figure 3A). Individual anti-N antibody dynamics and local regression according to participants’ reports regarding reinfection events are depicted in Figure 3B, showing significantly decreasing levels between the time of the first SARS-CoV-2 anti-N positive blood donation (0 months) and 9 months, spanning the first year after the primary infection (*p* < 0.0001) and significantly increasing anti-N levels from 9 until 36 months (*p* < 0.0001). So, in the long term, anti-N levels did not decline but significantly increased, reflecting the rising seroprevalence rate observed for the whole blood donor population of Salzburg (Figure 2). Interestingly, we did not find any significant difference regarding anti-N antibody levels for the different groups reporting various numbers of reinfection events. Anti-S antibody dynamics (Figure 3C) and in vitro antibody functionality (Figure 3D) increased over time for all participants with no significant difference between those reporting various numbers of reinfections. Calculating the number of reinfections based on the serological anti-N profile revealed a different profile of SARS-CoV-2 reinfection events: 47% revealed one rise in anti-N levels >30% compared to previous values suggesting one SARS-CoV-2 reinfection, 41% showed two such rises and 9% revealed more than two anti-N rises >30% (14 individuals with three anti-N increases >30%, 4 individuals with four anti-N increases >30%). So, according to their anti-N antibody profiles, 50% of the study participants had at least two SARS-CoV-2 reinfections. Furthermore, only 3% showed constantly low or decreasing anti-N levels, indicating no reinfection (Figure 3E,F). This contrasts with the 18% of participants, who reported no reinfections. Together, our data reveal a reinfection rate of 97% within the period observed. Dynamics of anti-S antibody levels (Figure 3G) and in vitro antibody functionality (Figure 3H) were comparable for all groups, which may be explained by the stimulation of anti-S-antibody production, not only through infection but also by vaccination.

Table 1 summarizes how many individuals showed an increase in SARS-CoV-2 anti-N antibodies >30% at specific points in time throughout the study. The highest number of increases (*n* = 107) was found at 18 months, which coincides with the arrival of the omicron variant in Europe. Even though the number of anti-N increases >30% declined between 18 and 24 months (from 107 to 65 cases), there was a substantial increase at 36 months again (*n* = 84), indicating that SARS-CoV-2 still caused a substantial number of infections in 2025. In sum, only 6 of the 204 participants did not show an increase in anti-N antibodies over the time observed.

### 3.3. Symptom Shift in SARS-CoV-2 Infection and Reinfections

The five most common symptoms reported within our study cohort for the primary infection were headache, hyposmia, body ache, fever and dysgeusia (Table 2). Regarding the symptoms of reinfections, we observed a change during the pandemic, particularly following the emergence of the omicron variant: ENT/respiratory symptoms, such as cough and sore throat, occurred substantially more frequently in cases of reinfection, while hyposmia and dysgeusia were reported less frequently compared to the primary infection (Table 2).

### 3.4. Anti-N and Anti-S Antibody Levels Show a Constant High Plateau Level Despite Vaccine Decay and a Decline in Vaccination Rates

**Figure 4:**
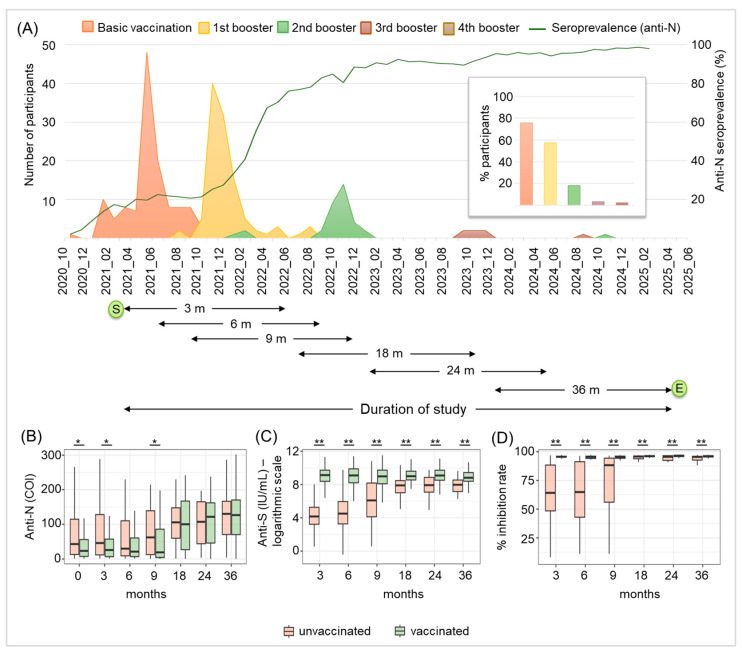
SARS-CoV-2 anti-N and anti-S antibody dynamics of vaccinated and unvaccinated participants. (**A**) Graphical representation of the period during which vaccinations against SARS-CoV-2 were administered (basic vaccination and booster vaccinations, as indicated) in comparison to the development of the SARS-CoV-2 anti-N seroprevalence rate among blood donors (dark green line), indicating the incidence of infection in the general blood donor population in Salzburg, Austria for the period observed. The duration of the study and the different collection periods for each time point of the study (3–36 months) are depicted below the time axis (time is indicated as month/year, m = months, S = start of the study, and E = end of the study). Anti-N (**B**) and anti-S (**C**) antibody dynamics and in vitro antibody functionality (**D**) of unvaccinated and vaccinated participants, as indicated. The time course shown covers the seropositive SARS-CoV-2 blood donation (0 months) to 36 months thereafter for anti-N antibodies (**B**) and 3–36 months after the seropositive SARS-CoV-2 blood donation for anti-S antibodies (**C**) and in vitro antibody functionality (**D**). * *p* < 0.01, ** *p* < 0.0001.

**Figure A1:**
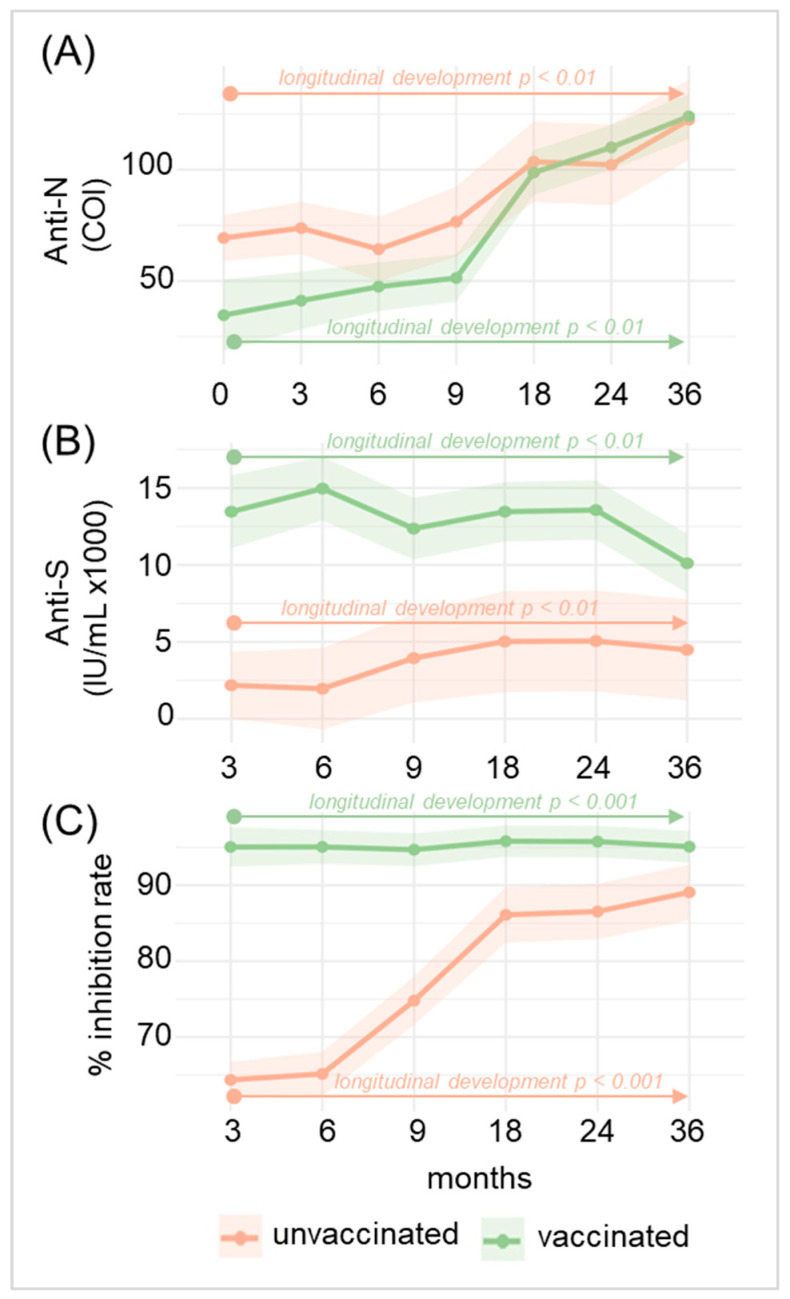
Longitudinal development of anti-N ((**A**), shown as cut-off index, COI) and anti-S ((**B**), IU/mL) antibody dynamics and (**C**) in vitro antibody functionality (indicated as % inhibition rate) over time for unvaccinated and vaccinated participants. *p*-values of longitudinal development are indicated in the graphs.

Next, we examined the anti-N and anti-S antibody dynamics, based on the presence or absence of specific vaccinations. Between October 2020 and December 2021, 78% of participants received the basic immunization consisting of at least one vaccination, with the majority being vaccinated in June 2021. A total of 57% received one booster vaccination, with most being vaccinated between December 2021 and February 2022. A further 18% had two booster vaccinations, with a peak for the second booster in November 2022. Only a few individuals received more than two booster vaccinations (3% received three, mostly in December 2023, and 3% received four, mainly in August 2024). Overall, the number of vaccinations administered decreased over time, while the SARS-CoV-2 anti-N seroprevalence continuously increased (Figure 4A). The numbers of vaccinated and unvaccinated individuals are summarized in Appendix A Table A1. Comparing anti-N antibody levels of vaccinated and unvaccinated individuals (Figure 4B), we found significant differences between the two groups at 0, 3 and 9 months: vaccinated individuals showed significantly lower anti-N antibody levels compared to those who were unvaccinated (*p* < 0.01). This difference could not be found in the later phases of the pandemic (18, 24 and 36 months). However, anti-N antibody levels generally rose significantly in both groups over the time observed (*p* < 0.01), reaching the highest mean cut-off index at 36 months (Figure 4B and Appendix A Figure A1A). As expected, we found a significant difference regarding anti-S antibody dynamics between unvaccinated and vaccinated participants, particularly for the early times: 3–9 months (*p* < 0.0001) (Figure 4C). Even though there was also a significant difference (*p* < 0.0001) for the later points in time (18–36 months) between vaccinated and unvaccinated individuals, the levels of anti-S antibodies in unvaccinated individuals also significantly rose (*p* < 0.01) over time. This is of particular interest, as no basic vaccinations were administered after July 2022 within our study cohort and the majority of booster vaccinations were administered until January 2023, so one year before the examination at 36 months. The decline in the vaccination rate later in the study can also be seen in the anti-S development of the vaccinated individuals, as there is a trend for decreasing anti-S concentrations (Figure 4C and Appendix A Figure A1B). Although the in vitro functionality of antibodies was significantly enhanced in both unvaccinated and vaccinated individuals during later periods when compared to earlier points in time (*p* < 0.001, Appendix A Figure A1C), vaccinated individuals revealed significantly higher (*p* < 0.0001) in vitro antibody functionality than unvaccinated participants (Figure 4D and Appendix A Figure A1C).

## 4. Discussion

The present study provides serological evidence that more than three years after a primary SARS-CoV-2 infection, and nearly five years after the onset of the pandemic, immunity to SARS-CoV-2 within the Austrian blood donor population has shifted from a state of declining, albeit persistent, specific antibody levels to antibody accumulation. Our study reveals that anti-N antibody levels also significantly increased during the post-pandemic period after May 2023, resulting in sustained high seroprevalence rates exceeding 90% from January 2023 onwards within the Austrian blood donor population. Antibody maturation might be a reason for rising antibody levels. However, it was previously demonstrated that six months following antigen contact, SARS-CoV-2 antibody maturation is finished [13]. Therefore, it is unlikely that antibody maturation is the main cause of the observed increases in anti-N levels in our long-term study, which determined the antibody dynamics for over 3 years after primary antigen stimulation. Our results are consistent with population-based sero-surveillance data from other European regions reporting sustained or rising antibody prevalence. Recently, long-lasting humoral anti-SARS-CoV-2 responses have been described in German blood donors with hybrid immunity due to infection and vaccination, despite reduced neutralization-based protection one year after infection [5]. Furthermore, in a four-year follow-up study from Switzerland, the proportion of anti-N-positive blood donors rose from 21.6% in spring 2021 to 83.9% at the end of 2022 and further to 95% in 2023, indicating a substantial increase during the omicron waves [16]. Similar findings were reported for the United Kingdom [17], the Netherlands [18] and also for the United States [11]. Like in our previous study, we used anti-N antibody dynamics in individual participants to determine reinfection rates. As anti-N antibodies are primarily produced after an infection, an increase in anti-N antibody levels of >30% compared to previous values was defined to indicate a reinfection event, as suggested previously [8,10]. While we found that 12% of participants showed constantly low or decreasing anti-N levels in our 24-month analysis [10], this proportion has decreased to 3% after 36 months. So, based on our longitudinal anti-N antibody level examination, 97% of our study participants experienced at least one reinfection and 50% had two or even more within 36 months of the initial SARS-CoV-2 infection. Furthermore, the serologically determined rates exceed self-reported reinfections by a factor of six (18% reported no reinfection, but only 3% actually did not have one), underscoring the high number of asymptomatic infections during the later phases of the pandemic. Our data corroborate the findings of other studies, revealing high numbers of asymptomatic SARS-CoV-2 reinfections [19,20,21]. The high prevalence of unrecognized reinfections emphasizes the limited accuracy of self-reported data and the importance of serological monitoring in reconstructing accurate exposure numbers. Together, our data and that from other studies suggest that immunity to SARS-CoV-2 is currently maintained through a dynamic equilibrium between waning immunity and recurrent boosting, which is mainly driven by reinfection events that frequently go unnoticed.

Furthermore, consistent with observations comparing mainly symptomatic infections with the SARS-CoV-2 variants delta and omicron [22,23,24,25], we found a change in symptom profiles between the initial SARS-CoV-2 infection and subsequent reinfections. While early infections mainly showed systemic and sensory symptoms such as fever, hyposmia and dysgeusia, later infections, particularly after the occurrence of the omicron variant, were mainly characterized by upper respiratory symptoms such as a sore throat and cough. These data suggest that, although reinfections are common, their clinical impact is increasingly mild and compatible with an endemic stage of virus circulation.

Our study further revealed that vaccinated participants initially exhibited lower anti-N levels than unvaccinated participants. These initially lower anti-N levels in vaccinated individuals likely reflect a combination of reduced infection incidence during early waves and the observation that infections occurring under strong anti-S immunity tend to induce weaker anti-N responses [26]. However, over time, anti-N antibody levels converged between unvaccinated and vaccinated individuals due to repeated antigenic exposures in both groups, which clearly suggests reoccurring SARS-CoV-2 infections as a main factor for the rising specific humoral response. As expected, anti-S antibodies and in vitro antibody functionality were lower for unvaccinated individuals at the beginning of our study. However, these levels also converged after the first year of the initial infection between vaccinated and unvaccinated individuals and remained at high, constant levels in later periods for both groups, pointing towards the effect of reinfection, rather than vaccination. Nevertheless, vaccinated individuals showed a significantly higher in vitro antibody functionality, most likely because of the accumulation of different antibodies obtained from infection and vaccination. Even though our findings align with reports regarding vaccinated individuals showing that hybrid immunity, derived from (multiple) infection(s) and vaccination(s), provides a robust and durable antibody response [3,9], our data clearly reveal that, in the post-vaccine era, repeated exposure to SARS-CoV-2 continues to reinforce immune responses, even in the absence of booster vaccination. The convergence of high anti-N seroprevalence (>90%), frequent but mild reinfections and the sustained plateau of the anti-N, anti-S and functional antibodies observed is consistent with a reinfection-stabilized endemic equilibrium of SARS-CoV-2 in Austria. This observed pattern closely matches theoretical models of coronavirus endemicity, in which infection-blocking immunity wanes but disease-reducing immunity persists, leading to a steady-state circulation [27]. Moreover, the recurrent boosting of humoral immunity resembles the long-term epidemiology of other endemic human coronaviruses such as HCoV-OC43 and HCoV-229E [28,29]. The antibody plateau might help to fight future SARS-CoV-2 virus variants, as repeated antigen exposure was demonstrated to expand the memory B-cell repertoire and promote a broader neutralization capacity [30,31].

The major strength of our study is the integration of a large cross-sectional dataset of more than 146,000 blood donations collected between June 2020 and March 2025 and the longitudinal observation of the SARS-CoV-2 antibody dynamics covering 36 months after the first SARS-CoV-2 infection. Furthermore, we examined anti-N and anti-S antibodies, also investigating in vitro antibody functionality for all points in time, ensuring analytical comparability. Nonetheless, there are several limitations: First, the cohort consists of healthy adults aged 18–70, limiting generalization and excluding older and younger individuals and individuals who were not admitted to blood donation for various health reasons. Therefore, this study may be subject to a healthy donor bias. Second, the inference of reinfections is based on serological rises in anti-N antibodies >30%, rather than viral sequencing; thus, transient assay fluctuations or unrecognized laboratory variability cannot be fully excluded. Third, regarding antibody screening, we cannot fully exclude a ceiling effect, so very high antibody levels may have approached the assay’s upper limits, potentially masking further increases. Furthermore, it is important to note that our conclusions were drawn from a pattern observed over 36 months from blood donors exposed to SARS-CoV-2 during the early days of the pandemic, and later, to different variants of the virus, with some of them causing milder disease courses [22,23,24,25]. Therefore, the observed high seroprevalence rates and pattern of mild reinfections may change in the future, due to new virus variants with different levels of infectivity, causing different disease courses and declining vaccination. Finally, vaccination timing and reinfections were self-reported, which may introduce recall bias.

## 5. Conclusions

Taken together, our findings show the SARS-CoV-2 anti-N and anti-S antibody dynamics of an Austrian blood donor population over a period of five years, revealing antibody persistence with waning levels during the early phases and reinfection-driven accumulation and a stable antibody plateau in the later, post-pandemic phases. Our data contribute evidence to the understanding of long-term SARS-CoV-2 humoral responses and point to the establishment of an endemic state of SARS-CoV-2 within the Austrian population.

## Figures and Tables

**Table 1:** Number and percentage of individuals with a SARS-CoV-2 anti-N increase >30% in the course of the study.

Time	Number Individuals with Anti-N Increase >30%	%
3 months	0	0.00
6 months	25	12.25
9 months	41	20.10
18 months	107	52.45
24 months	65	31.86
36 months	84	41.18

**Table 2:** Symptoms of the primary SARS-CoV-2 infection and reinfections. In addition, a time frame (month/year) when the primary infection and reported later reinfections occurred and the most common virus variant circulating during the period are listed.

Primary Infection, *n* = 204 Time: 12/2020–03/2022 Virus Circulating: SARS-CoV-2, Alpha, Beta, Delta and Omicron Variants			1st Reinfection, *n* = 96 Time: 08/2021–10/2023 Virus Circulating: Alpha, Beta, Delta and Omicron Variants			2nd Reinfection, *n* = 35 Time: 02/2022–02/2025 Virus Circulating: Delta, Omicron Variants			3rd Reinfection, *n* = 8 Time: 09/2022–08/2024 Virus Circulating: Omicron Variants		
	Symptoms	*n*		Symptoms	*n*		Symptoms	*n*		Symptoms	*n*
#1	Headache	72	#1	Cough	43	#1	Cough	16	#1	Sore throat	6
#2	Hyposmia	67	#2	Sore throat	37	#2	Sore throat	12	#2	Cough	5
#3	Body ache	65	#3	Headache	36	#3	Body ache	9	#3	Headache	5
#4	Fever	59	#4	Body ache	31	#4	Headache	8	#4	Body ache	4
#5	Dysgeusia	58	#5	Other symptoms **	31	#5	Hyposmia	8	#5	Other symptoms **	3
#6	Cough	49	#6	Fever	29	#6	Other symptoms **	5	#6	Shortness of breath	3
#7	Other symptoms *	45	#7	Shortness of breath	10	#7	Shortness of breath	4	#7	Fever	3
#8	Sore throat	32	#8	Hyposmia	6	#8	Dysgeusia	4	#8	Hyposmia	2
#9	Shortness of breath	20	#9	GI-tract issues	6	#9	Fever	3	#9	Dysgeusia	1
#10	GI-tract issues	16	#10	Dysgeusia	2	#10	GI-tract issues	1	#10	GI-tract issues	0

* Fatigue, rhinitis, back/chest pain, general malaise, feeling of coldness, allodynia, allodromy, chills, burning/tickling nose, sensitivity to light and night sweats. ** Fatigue, rhinitis, general malaise, allodynia, chest/back pain, hoarseness and skin rash.

## Data Availability

The data presented in this study are available upon request from the corresponding author, due to donor privacy.

## Abbreviations

The following abbreviations are used in this manuscript:

ACE2	Human Angiotensin-Converting Enzyme 2
ACOV2	Elecsys Anti-SARS-CoV-2 total antibody electrochemiluminescence immunoassay
COVID-19	Coronavirus Disease 2019
ECDC	European Center for Disease Prevention and Control
ECLIA	Electrochemiluminescence Immunoassay
SARS-CoV-2	Severe Acute Respiratory Syndrome Coronavirus 2
WHO	World Health Organization

## Appendix A

**Table A1:** Number of participants (*n*) unvaccinated or vaccinated at a specific time during the study.

Point in Time	Unvaccinated (*n*)	Unvaccinated (%)	Vaccinated (*n*)	Vaccinated (%)
0 months	147	72.1	57	27.9
3 months	113	55.4	91	44.6
6 months	73	35.8	131	64.2
9 months	61	29.9	143	70.1
18 months	46	22.5	158	77.5
24 months	46	22.5	158	77.5
36 months	46	22.5	158	77.5
